# Genomic and Bioinformatic Characterization of Mouse Mast Cells (P815) Upon Different Influenza A Virus (H1N1, H5N1, and H7N2) Infections

**DOI:** 10.3389/fgene.2019.00595

**Published:** 2019-06-21

**Authors:** Caiyun Huo, Hongping Wu, Jin Xiao, Di Meng, Shumei Zou, Ming Wang, Peng Qi, Haiyan Tian, Yanxin Hu

**Affiliations:** ^1^Key Laboratory of Animal Epidemiology of Ministry of Agriculture, College of Veterinary Medicine, China Agricultural University, Beijing, China; ^2^Key Laboratory of Veterinary Bioproduction and Chemical Medicine of the Ministry of Agriculture, Zhongmu Institutes of China Animal Husbandry Industry Co., Ltd, Beijing, China; ^3^Key Laboratory for Medical Virology, National Health and Family Planning Commission, Collaboration Innovation Center for Diagnosis and Treatment of Infectious Diseases, Chinese Center for Disease Control and Prevention, National Institute for Viral Disease Control and Prevention, Beijing, China

**Keywords:** cellular functions, differentially expressed genes, influenza A virus, mast cells, signaling pathways

## Abstract

Influenza A virus (IAV) is a segmented negative-stranded RNA virus that brings a potentially serious threat to public health and animal husbandry. Mast cells play an important role in both the inherent and adaptive immune response. Previous studies have indicated that mast cells support the productive replication of H1N1, H5N1, and H7N2. To date, the distinct molecular mechanism behind the pathogenesis in mast cells among the three different viruses has been poorly understood. In this study, we investigated the genomic profiles in detail and the dynamic change of genomes regulated by different subtypes of IAV in mouse mast cells using microassays. Compared with any two of the three IAV-infected groups, many more differentially expressed genes (DEGs), cellular functions, and signaling pathways were confirmed in H1N1 or H7N2 group, with the H7N2 group showing the highest levels. However, few DEGs were detected and various cellular functions and signaling pathways were dramatically suppressed in the H5N1 group. With an in-depth study on the H1N1 and H7N2 groups, we demonstrated the essential role of the 5-HT signaling pathway and the cyclic guanosine monophosphate (cGMP)/protein kinase G (PKG) signaling pathway, which were preferentially activated in P815 cells infected by H1N1, and the crucial role of the HIF-1 signaling pathway that was preferentially activated in P815 cells infected by the H7N2 virus. Furthermore, real-time quantitative polymerase chain reaction (RT-qPCR) results showed significantly increased mRNA levels of 5-HT and PKG in H1N1-infected P815 cells and increased HIF-1 in H7N2-infected P815 cells. In addition, exosomes were preferentially secreted from H1N1-infected or H7N2-infected P815 cells and are potentially pivotal in innate immunity to fight IAV infection. This study provides novel information and insight into the distinct molecular mechanism of H1N1, H5N1, and H7N2 viruses in mast cells from the perspective of genomic profiles.

## Introduction

Influenza A virus (IAV) is a segmented negative-stranded RNA virus that brings a potentially serious threat to public health and animal husbandry. Based on the surface hemagglutinin (HA) and neuraminidase (NA) proteins of the viruses, IAVs are classified into 18 H subtypes (H1-H18) and 11 N subtypes (N1-N11) (Fouchier et al., 2005; Tong et al., 2013). Waterfowl and coastal birds have been considered the reservoir hosts for IAVs, and some IAVs that originated from wild birds have established stable transmission profiles among birds, pigs, and humans. The genome of IAVs consists of eight segments that encode 17 viral proteins to perform diverse biological functions (Vasin et al., 2014). IAV-induced acute lung injury or even acute respiratory distress syndrome is a major cause of mortality by pandemic influenza and highly pathogenic avian H5N1 infection. In terms of the pathogenic mechanism, the over-secretion of inflammatory cytokines (“cytokine storm”) induced by the immune or inflammatory response during viral infection is considered a major factor (Tisoncik et al., 2012).

Mast cells, a type of immune cells with numerous allochromatic granules, originate from hematopoietic stem cells and can be transferred to target tissues after early differentiation and eventually reach maturation (Galli et al., 2011). Mature mast cells are mainly concentrated in the skin, airways, and digestive tracts and play an important role in both the inherent and adaptive immune response (Henz et al., 2001; Galli et al., 2005). Mast cells can participate in the anti-parasitic response, autoimmunity diseases, wound healing, and tissue remodeling during chronic asthma (Abraham and St John, 2010). Mast cells are also involved in inflammatory responses such as the release of a variety of inflammatory cytokines and chemokine responses to bacterial and viral infection (Marshall et al., 2003). Our previous studies have demonstrated that mast cells support the productive replication of H1N1 (A/WSN/33), H5N1 (A/chicken/Henan/1/04), and H7N2 (A/chicken/Hebei/2/02) (Liu et al., 2014; Meng et al., 2016). All the three viruses could induce mast cell apoptosis and pro-inflammatory cytokine response. Furthermore, toll-like receptor 3 (TLR3) plays a key role in the expression of pro-inflammatory cytokines in IAV-infected mast cells. To date, there has been little research that focuses on explaining the distinct molecular mechanism behind the pathogenesis in mast cells among the three different viruses by comparison at the whole genomic level.

Identification and quantification of differentially expressed genes (DEGs) can be performed by gene ontology (GO) enrichment analysis and the Kyoto Encyclopedia of Genes and Genomes (KEGG) analysis using a gene chip assay. GO consists of three parts including molecular function (MF), biological process (BP), and cellular component (CC), and each of them gets various GO terms. Therefore, the information regarding the relationships between DEGs and cellular functions can be obtained (Gusev, 2008). KEGG analysis is regarded as a pathway analysis to search for the cellular pathways as well as the relationships among them. According to the pathway enrichment terms of DEGs, it is helpful to acquire the crucial cellular pathways that are associated with DEGs (Kanehisa et al., 2008). To date, these techniques have been widely used in many fields of life science and can be used to detect gene expression levels and explore gene regulation mechanisms (Sugiura et al., 2011). Researchers have adapted this gene expression analysis and have found the genes coding for chemokines are also strongly and, in many cases, continuously induced in H5N1-infected ferrets (Cameron et al., 2008). However, a systematic comparison of the genomic changes activated by different subtypes of IAV infection has yet to be explored.

To gain a deeper understanding of the relationship between mast cells and the various IAV viruses and to explore the distinct molecular mechanism behind the pathogenesis in mast cells among the three different viruses, we used microarray analysis to obtain unbiased quantitative information on the relative gene abundance in mouse mast cells (P815) during infection by human H1N1, avian H5N1, or H7N2 IAV at 12 h. In particular, we aimed to provide insights into the mechanistic differences in host responses induced by these three subtypes of IAV viruses.

## Materials and Methods

### Viruses and Cell Culture

The H1N1 (A/WSN/33), H5N1 (A/Chicken/Henan/1/04), and H7N2 (A/Chicken/Hebei/2/02) viruses were isolated, and virus titers were determined as described previously (Huo et al., 2018). The mouse mastocytoma cell line P815 and MDCK cells were provided and cultured as previously described (Hu et al., 2012). All experiments with IAV were conducted in a biosafety level 3 containment laboratory approved by the Ministry of Agriculture of China.

### *In vitro* Viral Infection

Cells were seeded and viral infection was taken at a multiplicity of infection (MOI) of 1 for 12 h as previously described (Meng et al., 2016).

### RNA Extraction

Total RNA was extracted from cells after 12 h post-infection using Trizol reagents according to the manufacturer’s recommended protocol.

### Microarray Analysis

Mouse gene expression was examined with the GeneChip Mouse Genome 4300 2.0 array (Affymetrix). Briefly, first strand cDNA was reverse transcribed using a First Strand Enzyme Mix (Affymetrix) and T7 Oligo (dT) Primer according to the manufacturer’s instructions. The reaction was run on a PCR instrument (PTC-225, MJ). Second Strand Enzyme Mix (Affymetrix) was then used to convert the DNA-RNA hybrid into second strand cDNA, and double-stranded DNA was synthesized. *In vitro* transcription and synthesis of cRNA was performed by using T7 Enzyme Mix (Affymetrix), which was added with biotin according to the manufacturer’s instruction. During the process of cRNA purification, cRNA was purified by magnetic beads, impurities such as salt and enzyme were removed and cRNA was quantified. cRNA was then fragmented into the appropriate size to facilitate hybridization. Finally, cRNA was hybridized to the GeneChip array according to the manufacturer’s protocol. The GeneChips were finally washed and stained using the GeneChip Fluidics Station 450 (Affymetrix) and then scanned with the GeneChip Scanner 3000 (Affymetrix).

### Sequence Database Search and Data Analysis

The original microarray data were uploaded into NCBI GEO repository and the GEO accession number was GSE129623. The raw mass data were processed for the gene data analysis using the normalized FPKM values obtained by DEGseq. Based on the negative binomial distribution model, heterogenetic analysis among samples was obtained by controlling the Fold-change (FC) and *p* statistical methods. The screening conditions of this study were |log2FC| > 1 and *p* < 0.05. An analysis of variance (ANOVA), using the Benjamini-Hochberg multiple testing correction, was performed to identify genes significantly differentially expressed (*p* < 0.05) in response to virus infection. Significantly differential expressed genes with fold change ≥2 were then merged into a gene list for further gene ontology (GO) and pathway analysis.

### Bioinformatics and Statistical Analyses

The GO and KEGG pathway analyses on the DEGs were performed using DAVID Bioinformatics Resources 6.8^1^. The threshold of significantly enriched genes was determined according to the enrichment degree (count ≥ 2) and correct *p* (Benjamini) (*p* ≤ 0.05). The default parameters (hypergeometric algorithm, Benjamini-Hochberg multiple testing correction) was performed to take over-representation analyses. Results with *p* of <0.05 after multiple testing corrections were considered statistically significant. Results with only a single GO term or pathway were not considered. The heatmaps of DEPs were achieved with the pheatmap packages from R. The DEGs related to IAV were used to predict genegene interactions network by using STRING online^2^ and visualized by Cytoscape software.

### Real-Time Quantitative Polymerase Chain Reaction Analysis

The procedures were performed as previous described (Liu et al., 2014). The primers for genes were as follows: 5-HT (2A) (F: 5′-ATGTGGTGCCCATTCTTCAT-3′, R: 5′-CGGCTGAGGAGAGATAACCA-3′); PKG II (F: 5′-GCTGTGTCTGCTCAGGTATTAGGGGC-3′, R: 5′- GCCAGCTCGCAGAGCACTCCCGCAG-3′); HIF-1α (F: 5′-GGACAAGTCACCACAGGACA-3′, R: 5′-GGGAGAAAATCAAGTCGTGC-3′).

### Exosome Isolation and Western Blotting

Exosomes from cell culture supernatants were isolated and characterized by Western blotting as described previously (Welton et al., 2010). Here, the concentration of CD63 antibody (System Biosciences) was 1:1,000.

### Enzyme-Linked Immuno Sorbent Assay

Exosomes quantification was taken by detecting the concentration of CD63 through enzyme-Linked Immuno Sorbent Assay (ELISA) kits according to the manufacturer’s instructions.

## Results

### Identification and Quantification of Differentially Expressed Genes by Using Affymetrix Microarray

**Figure 1:**
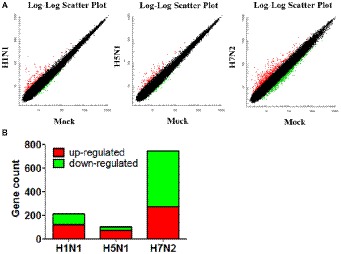
Identification and quantification of differentially abundant genes between IAV-infected P815 cells and uninfected cells. **(A)** Scatter plots of gene abundance ratio in H1N1vsMock, H5N1vsMock, and H7N2vsMock, respectively. **(B)** Bar chart showing the up-regulated DEPs (red) and down-regulated DEGs (green) in P815 cells infected with different subtypes of IAV (H1N1vsMock, H5N1vsMock, and H7N2vsMock), respectively.

We used the Affymetrix GeneChip Mouse Genome 4300 2.0 array to compare the global gene expression profiles of mouse mastocytoma cell line P815 infected with H1N1, H5N1, H7N2 viruses and mock-infected control cells at 12 h post-infection. By detecting and quantifying unique genes, we found that 216 genes were acquired in line with biological statistics in H1N1 compared with Mock. At the same time, there were 101 genes in H5N1 compared with Mock as well as 745 genes in H5N1 compared with Mock, respectively (Figure 1A). Further analysis, the numbers of genes that up- and down-regulated their abundance were summarized in this study using a cutoff ratio of >2 or < 0.5 (*p* < 0.05). As shown as in Figure 1B, H1N1 virus induced 121 significantly up-regulated genes and 95 significantly down-regulated genes as well as 6 non-coding RNAs (ncRNAs) in mast cells (H1N1vsMock). There were 101 abundant genes including 74 significantly up-regulated genes and 27 significantly down-regulated genes in cells infected by H5N1 virus (H5N1vsMock) at 12 h. As for H7N2 virus, the up-regulation of 274 genes, the down-regulation of 471 genes and 17 ncRNAs were confirmed in P815 cells at 12 hpi following H7N2 infection (H7N2vsMock).

**Figure 2:**
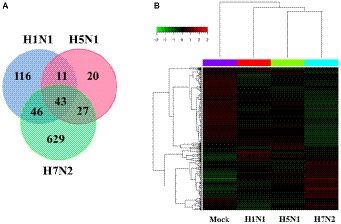
Identification and quantification comparison of differentially abundant genes in P815 cells infected with different subtypes of IAV. **(A)** Venn diagrams showing the overlap of DEGs in P815 cells infected with three subtypes of IAV, respectively. **(B)** Heat map presenting the screened DEGs.

In addition, we also compared and analyzed the differentially expressed genes (DEGs) from the obtained genes (Figure 2A). We summarized 116 DEGs uniquely in H1N1-infected P815 cells, 20 DEGs uniquely in H5N1-infected P815 cells and also 629 DEGs uniquely in H7N2-infected P815 cells, respectively. At the same time, 43 differentially abundant genes were commonly obtained from three groups. As shown in Figure 2B, the heat map of DEGs showed that these DEGs were all significantly distributed in P815 cells infected with the corresponding influenza virus, respectively. Taken together, identification of a larger number of differentially abundant genes in P815 cells following different subtypes of IAVs suggested that infection with various IAV strains resulted in significantly changes in the global genome of P815 cells.

### Functional Annotations and Bioinformatic Characterization of Unique DEGs in P815 Cells Infected With H1N1 Virus Compared With H5N1 Virus

**Figure 3:**
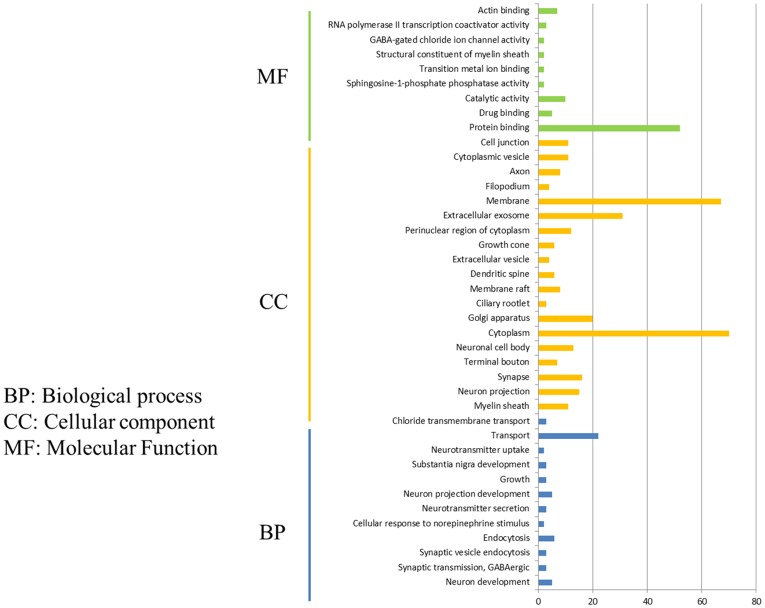
Functional annotations and bioinformatic characterization of unique DEGs in P815 cells infected with H1N1 virus compared with H5N1 virus. The GO biological process terms, GO cellular component terms, and GO molecular function terms of unique DEGs in P815 cells infected with H1N1 virus and H5N1 virus were analyzed, respectively. Green bar chart represented biological classification of the IAV-regulated unique DEGs in P815 cells. Orange bar chart represented cellular classification of the IAV-regulated unique DEGs in P815 cells. Blue bar chart represented molecular classification of the IAV-regulated unique DEGs in P815 cells.

After comparison of DEGs in P815 cells with challenge of H1N1 virus and H5N1 virus, we demonstrated that the 162 unique DEGs were identified in H1N1-infected P815 cells and 47 unique DEGs were identified in H5N1-infected P815 cells, respectively. Then, GO enrichment analysis and KEGG-pathway analysis were conducted using the DAVID online analysis system for the probe ID of these screened DEGs. As shown in Figure 3, 141 DEGs enriched in the 190 GO biological process terms, 150 DEGs enriched in the 103 GO cellular component terms, and 133 DEGs actively participated in the 112 GO molecular function terms could be present in H1N1-infected P815 cells. According to the standard screening of DEGs count ≥ 2 and *p* < 0.05, 12 biological process items, 20 cell component items, and 10 molecular function items were significantly enriched, which mainly be involved in the signaling pathways such as transport, endocytosis, cytoplasm, membrane, exosomes, protein binding, catalytic activity, RNA polymerase II transcription coactivator activity terms, and so on. In addition, KEGG-pathway analysis showed that 63 DEGs were enriched in the 92 KEGG-pathway items, which 20 of these KEGG-pathway items were significantly enriched and were associated with the 5-hydroxytryptamine (5-HT) synapses, endocytosis, cGMP-PKG signaling pathway, neuron-related morphine addiction, glutamate synapses, and gap junctions.

By taking the GO enrichment analysis and KEGG-pathway analysis of 47 unique DEGs in H5N1-infected P815 cells, we found that 30 DEGs were enriched in the 22 GO biological process terms, 30 DEGs were enriched in the 39 GO cellular component terms and 28 DEGs actively participate in the 80 GO molecular function terms. Furthermore, one biological process items related to neuron development, one cell component items for cytoplasmic component, and one molecular function items for the activity of transferase and transfer acyl were significantly enriched. Notably, though nine DEGs were enriched in the two KEGG-pathway items, no KEGG-pathway item was significantly enriched. Identification of a larger number of DEGs in H1N1vs H5N1 suggested that infection with H1N1 strains resulted in more changes in the global genome of P815 cells than did infection with H5N1 virus.

### Functional Annotations and Bioinformatic Characterization of Unique DEGs in P815 Cells Infected With H1N1 Virus Compared With H7N2 Virus

**Figure 4:**
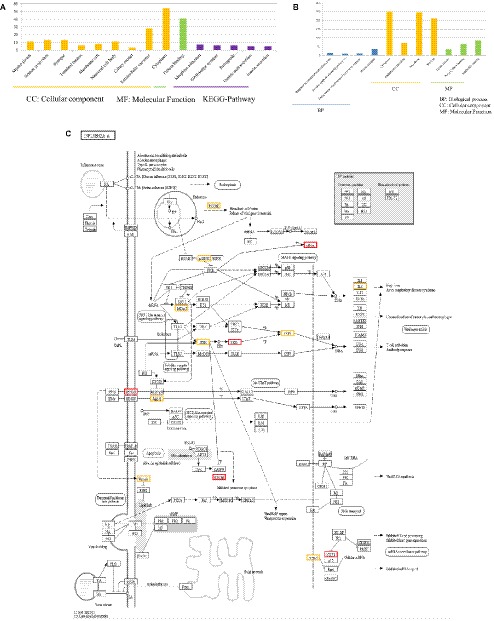
Functional annotations and bioinformatic characterization of unique DEGs in P815 cells infected with H1N1 virus compared with H7N2 virus. **(A,B)** The GO biological process terms, GO cellular component terms and GO molecular function terms of unique DEGs in P815 cells infected with H1N1 virus and H7N2 virus were analyzed, respectively. Orange bar chart represented cellular classification of the IAV-regulated unique DEGs in P815 cells. Green bar chart represented molecular classification of the IAV-regulated unique DEGs in P815 cells. Blue bar chart represented biological classification of the IAV - regulated unique DEGs in P815 cells. Purple bar chart represented KEGG pathways of the IAV- regulated unique DEGs in P815 cells. **(C)** The regulation changes of DEGs involved in IAV signaling pathway. Red represented up-regulated DEGs. Yellow represented down-regulated DEGs.

By comparing DEGs in P815 cells with challenge of H1N1 virus and H7N2 virus, we identified the 127 unique DEGs identified in H1N1-infected P815 cells and 162 unique DEGs identified in H7N2-infected P815 cells. Likewise, we also took GO enrichment analysis and KEGG-pathway analysis. As shown in Figure 4A, in H1N1-infected P815 cells, there were 107 DEGs enriched in the 155 GO biological process terms, 113 DEGs enriched in the 89 GO cellular component terms, and 100 DEGs actively participated in the 90 GO molecular function terms. Based on the standard screening of DEGs count ≥ 2, *p* ≤0.05 and Benjamini correction ≤ 0.05, no biological process terms and one cellular component terms for protein binding were significantly enriched. Nine biological process items were found to be significantly enriched, which were obviously classified into the cytoplasm, extracellular exosomes, neuron projection, protein binding and so on. In terms of KEGG-pathway, we found 5 significant enrichment pathways among 48 DEGs from our results on KEGG pathways database based on DAVID, which mainly participated in the GABAergic synapse, morphine addiction, retrograde, gastric acid secretion, and insulin secretion.

By contrast, the functions of unique DEGs in H7N2-infected P815 cells were different from that in H1N1-infected P815 cells. As shown in Figure 4B, we could see that 505 DEGs in the 761 GO biological process terms, 543 DEGs in the 235 GO cellular component terms, and 495 DEGs in the 265 GO molecular function terms were enriched in H7N2-infected P815 cells, respectively. Among them, four biological process terms which involved in the response to endoplasmic reticulum stress, intrinsic apototic signaling pathway, endoplasmic reticulum unfolded protein response, and protein response were significantly enriched. Also, four cellular component terms for protein binding were significantly enriched, which were mainly involved in cytoplasm, endoplasmic reticulum, cell membrane, and nucleus. Besides, biological process items for the nucleic acid binding, ligase activity, and Poly(A) RNA binding were significantly enriched. At the same time, there were 227 enriched in the KEGG-pathway terms, and 27 terms of them were enriched significantly. These significant KEGG-pathway terms were obviously classified into the protein processing in endoplasmic reticulum, insulin resistance, influenza A infection, viral carcinogenesis, tumor necrosis factor (TNF), phosphatidylinositol, and adenosine monophosphate activated protein kinase (MAPK) signaling pathways. As shown in Figure 4C, the analysis of related DEGs participated in influenza A infection signaling pathway could show that IFNAR2, PKB, eIF, NXF1, and GSK38 genes were up-regulated. However, the genes of PKC, p58IPK, MDA5, P13K, IRF3, MDA5, IL-6, Nup98, and Jak1 were all down-regulated. All these could be used as candidate genes to study the relationships between influenza A virus and hosts. Taken together, these results suggested that infection with H7N2 strains resulted in more gene changes in the global genome of P815 cells than did infection with H1N1 virus.

### Functional Annotations and Bioinformatic Characterization of Unique DEGs in P815 Cells Infected With H5N1 Virus Compared With H7N2 Virus

Comparison of DEGs in P815 cells with infection of H5N1 virus and H7N2 virus could show that the 31 unique DEGs were identified in H5N1-infected P815 cells and 162 unique DEGs were identified in H7N2-infected P815 cells. Eleven GO biological process terms were enriched with 162 DEGs, 15 GO cellular component terms were enriched with 20 DEGs, and 11 GO molecular function terms were enriched with 17 DEGs, respectively. However, none of these terms showed significant enrichment. Although three DEGs actively participated in a metabolic KEGG-signaling pathway terms, the result of this signaling pathway was false positive according to the standard screening of DEGs count ≥ 2, *p* ≤ 0.05.

**Figure 5:**
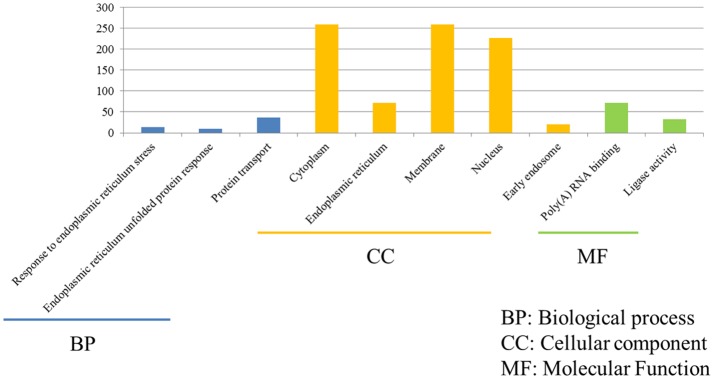
Functional annotations and bioinformatic characterization of unique DEGs in P815 cells infected with H5N1 virus compared with H7N2 virus. The GO biological process terms, GO cellular component terms and GO molecular function terms of unique DEGs in P815 cells infected with H7N2 virus were analyzed compared with H5N1 virus. Blue bar chart represented biological classification of the IAV-regulated unique DEGs in P815 cells. Orange bar chart represented cellular classification of the IAV-regulated unique DEGs in P815 cells. Green bar chart represented molecular classification of the IAV-regulated unique DEGs in P815 cells.

The functions of 675 unique DEGs in H7N2-infected P815 cells were also distinct to some extent. As shown in Figure 5, there were 526 DEGs enriched in the 783 GO biological process terms, 570 DEGs enriched in the 245 GO cellular component terms, and 517 DEGs enriched in the 274 GO molecular function terms in H7N2-infected P815 cells, respectively. With the standard screening of DEGs count ≥ 2, *p* ≤ 0.05 and FDR ≤ 0.05, three biological process terms, five cellular component terms, and nine biological process terms were significantly enriched, which were obviously classified into the response to endoplasmic reticulum stress, endoplasmic reticulum unfolded protein response, protein transport, cytoplasm, endoplasmic reticulum, membrane, nucleus, early endosome, Poly(A) RNA binding, and ligase activity. In terms of KEGG-pathway, we found 26 significant enrichment pathways among 236 DEGs from our results on KEGG pathways database based on DAVID, which mainly participated in such as he protein processing in endoplasmic reticulum, insulin resistance, hypoxia-inducible factor (HIF), influenza A infection, viral carcinogenesis, tumor necrosis factor (TNF), and adenosine monophosphate activated protein kinase (MAPK)-signaling pathways. The analysis of related DEGs participated in influenza A infection signaling pathway could show that IFNAR2, PKB, eIF, NXF1, and GSK38 genes were up-regulated. Meanwhile, the genes of PKC, p58IPK, MDA5, P13K, IRF3, MDA5, IL-6, Nup98, and Jak1 were all down-regulated. All of these could be used as candidate genes to study the relationships between influenza A virus and hosts. Taken together, these results suggested that infection with H7N2 strains resulted in more gene changes in the global genome of P815 cells than did infection with H5N1 virus.

### Functional Annotations and Bioinformatic Characterization of Common DEGs in P815 Cells With Three Different Subtypes of IAV Infection

**Figure 6:**
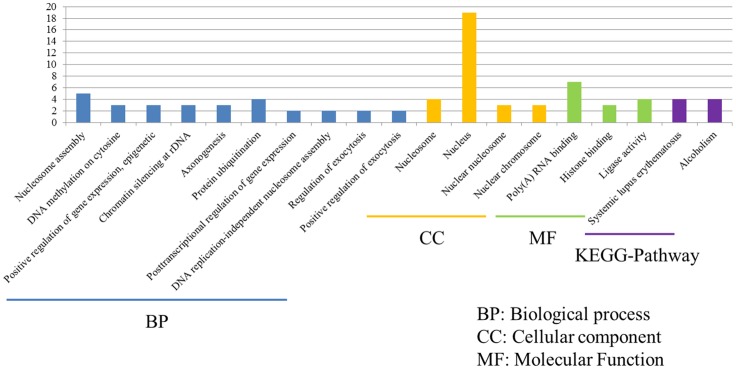
Functional annotations and bioinformatic characterization of common DEGs in P815 cells with IAV infection. The GO biological process terms, GO cellular component terms and GO molecular function terms of common DEGs in P815 cells with IAV infection. Blue bar chart represented biological classification of the IAV - regulated unique DEGs in P815 cells. Orange bar chart represented cellular classification of the IAV-regulated unique DEGs in P815 cells. Green bar chart represented molecular classification of the IAV-regulated unique DEGs in P815 cells.

Despite P815 cells were challenged with different subtypes of IAV (H1N1/H5N1/H7N2), there were also 43 common DEGs that could be identified in infected cells. Among these common DEGs, there were 33 DEGs enriched in the 38 GO biological process terms, 33 DEGs enriched in the 34 GO cellular component terms, and 33 DEGs actively participated in the 23 GO molecular function terms (Figure 6). Based on the standard screening of DEGs count ≥ 2 and *p* ≤ 0.05, 10 biological process terms, 4 cellular component terms and 9 biological process items were significantly enriched, which were obviously classified into the nucleosome assembly, DNA methylation on cytosine, positive regulation of gene expression, epigenetic, chromatin silencing at rDNA, axonogenesis, protein ubiquitination, posttranscriptional regulation of gene expression, DNA replication-independent nucleosome assembly, regulation of exocytosis, positive regulation of exocytosis, nucleosome, nucleus, nuclear chromosome, Poly(A) RNA binding, histone binding and ligase activity. In terms of KEGG-pathway, we found 5 significant enrichment pathways among 48 DEGs from our results on KEGG pathways database, which mainly participated in the systemic lupus erythematosus and alcoholism. Furthermore, the genes involved in these two signaling pathways were all up-regulated genes and belonged to the histone family genes, including H3F3b, Hist1h2bc, Hist1h3a, and Hist1h4a, indicating that histone family genes could play a pivotal role in regulated signaling pathways.

### The Distinct of Gene Expressions of 5-HT, PKG, and HIF-1 in P815 Cells Among Three Subtypes of IAVs

**Figure 7:**
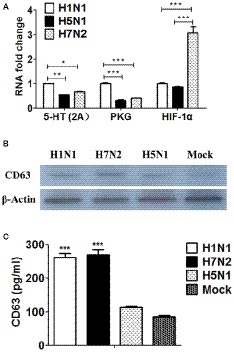
The distinct of expressions of 5-HT, PKG, and HIF-1 as well as exosomes in P815 cells among three subtypes of IAVs. **(A)** The mRNA levels of 5-HT, PKG and HIF-1 in P815 cells following H1N1, H5N1, and H7N2 infection were determined by real-time PCR (*N* = 3); *p < 0.05, **p < 0.01, ***p < 0.001. **(B)** The protein levels of CD63 in P815 cells following H1N1, H5N1 and H7N2 infection were detected by western blotting. **(C)** The protein levels of CD63 in P815 cells following H1N1, H5N1, and H7N2 infection were quantitatively detected by ELISA (*N* = 3); ***p < 0.001.

Based on the genomic results, we surprisingly found that DEGs were significantly enriched in the terms of 5-HT signaling pathway and cGMP/PKG signaling pathway in P815 cells following H1N1 infection. Besides, DEGs were significantly enriched in the terms of HIF-1 signaling pathway in P815 cells during H7N2 infection. In order to further validate the results, the real-time quantitative polymerase chain reaction (RT-qPCR) was used to detect the gene expression of 5-HT, PKG and HIF-1 in P815 cells among three subtypes of IAVs, respectively. As shown in Figure 7A, H1N1 group showed the significantly increased mRNA levels of 5-HT (2A) and PKG II genes than any other two groups. In addition, the expression of HIF-1α was the much highest in H7N2-infected P815 cells among three groups. In terms of H5N1 group, all of three genes were lower compared to H1N1 group or H7N2 group, which was in accordance with above microassays that few cellular functions and signaling pathways were found in H5N1 groups. Taken together, the results suggested that the gene expressions of 5-HT, PKG and HIF-1 in P815 cells were distinct among three subtypes of IAVs.

### The Distinct of Exosomes in P815 Cells Among Three Subtypes of IAVs

According to the results of above genomic profiles, exosomes were present in P815 cells following H1N1 and H7N2 infection. To further confirm whether exosomes were secreted and play roles in infected P815 cells, differential ultracentrifugation method was used to isolate the exosomes from P815 cells infected with three subtypes of IAVs, respectively. Due to that CD63 belongs to a membrane protein that located on the surface of exosomes, then the protein expression of CD63 was measured. As shown in Figure 7B, the mock P815 cells could secret exosomes, which the protein size was about 53 kDa. Compared to mock group, H1N1 and H7N2 viruses could result in the highly secretion of exosomes, respectively. However, the levels of exosomes in H5N1 group were the similar to that in mock group, indicating the lowest levels of exosomes in P815 cells following H5N1 infection among three subtypes of IAVs. Moreover, the concentration of CD63 was also quantitatively detected by using ELISA kits. As shown in Figure 7C, the protein levels of CD63 in H1N1 group and H7N2 group were significantly higher than that in H5N1 group or mock group, which were consistent with the results of western blotting. Together, the results verified that exosomes were preferentially secreted from H1N1 or H7N2-infected P815 cells rather than H5N1-infected P815 cells.

## Discussion

Genomic and bioinformatic analyses have been used to study dynamic alterations in global gene profiles after IAV infection of various cell types using microassays (Cameron et al., 2008). Recently, mast cells have been found to play an essential role during IAV infection (Boyce, 2007; Galli et al., 2011; da Silva et al., 2014; Liu et al., 2014; Meng et al., 2016). Immune mediators such histamine, proteases, leukotrienes, inflammatory cytokines, and antiviral chemokines can also be directly released by mast cells after IAV (H1N1, H5N1 and H7N2) infection. Here, we focused on how the quantities of mouse mast cells genes may be regulated by different subtypes of IAV virus infections, and performed an in-depth analysis of the relative functional activities of these various genes. Thus, the cellular dynamic processes of DEGs modulated by different IAV stains in P815 cells were researched in this study. These data will help us further understand the molecular mechanisms of IAV infection in mast cells, pathogenesis and host defense.

In our study, we specifically assessed the DEGs in mouse P815 cells and the related cellular functions and signaling pathways in the three subtypes of IAVs. Among these viruses, we have confirmed that the amount of enrichment of DEGs was dramatically different, with H7N2 groups showing the highest amount, while H5N1 groups showed the lowest amount. According to the results of the GO and KEGG-pathway analysis, we also demonstrated that many more cellular functions and signaling pathways were actively triggered in P815 cells following H7N2 or H1N1 infection, while distinct differences continued to exist between these two groups. By contrast, few cellular functions and signaling pathways were found in H5N1 groups, indicating a variety of biological mechanisms were largely suppressed in P815 cells during H5N1 infection. Taken together, our genomic and bioinformatic results illustrate the distinct molecular mechanism of H1N1, H5N1, and H7N2 viruses in mast cells. In addition, we found cellular functions and signaling pathways that uniquely participated in P815 cells during H1N1 or H7N2 infection. Well-known cellular functions and signaling pathways include protein binding, nucleotide binding, TNF signaling pathway, and MAPK signaling pathways. To our knowledge, we are the first group to report additional new cellular functions and signaling pathways in mast cells following IAV infection.

### Essential Role of the 5-HT Signaling Pathway and cGMP/PKG-Signaling Pathway in P815 Cells Infected by H1N1 Infection

Mast cells are known to contain abundant basophilic keratohyalin granules and mediators such as histamine, 5-hydroxytryptamine (5-HT), heparin, prostaglandin (PGD), and interleukin and vascular endothelial growth factor (VEGF) (Marshall, 2004; Boyce, 2007). Among them, 5-HT is one of the most extensively examined neurotransmitters in the central nervous system and also present in a variety of peripheral tissues in constituents of the immune system (Dimitrov et al., 2017; Wu et al., 2019). Previous studies have shown that 5-HT released by macrophages plays an essential role in T-cell and natural killer (NK)-cell activation, delayed-type hypersensitivity responses, production of chemotactic factors, and natural immunity (Mossner and Lesch, 1998). In addition, 5-HT has been shown to play a role in the granules of mast cells, which affects degranulation of mast cells and expression of inflammatory factors (Benditt et al., 1955; Wu et al., 2015; Dimitrov et al., 2017). In this study, the 5-HT signaling pathway with *p* ≤ 0.05 was preferentially and significantly modulated by DEGs induced by the H1N1 virus in P815 cells. RT-qPCR further confirmed the significantly increased gene levels of 5-HT in P815 cells infected by H1N1. However, the avian H5N1 or H7N2 virus did not trigger the 5-HT signaling pathway and did not improve the gene expression of 5-HT in P815 cells. Thus, our results demonstrate for the first time that the 5-HT signaling pathway has an essential role in P815 cells following H1N1 infection.

The cyclic guanosine monophosphate (cGMP)/protein kinase G (PKG) signaling pathway is an important intracellular signal transduction pathway with a pivotal role in various cells (Lohmann et al., 1997). The upstream molecules are mainly nitric oxide (NO) and guanylate cyclase (GC), while its downstream molecules are mainly composed of phosphodiesterase (PDE), protein kinase G (PKG), and gated ion channel. cGMP not only activates PKG to trigger a series of downstream reactions mediated by phosphorylation of related substrates of serine/threonine but also inhibits PDE3 to indirectly promote intracellular cyclic adenosine monophosphate (cAMP) and thus amplify the biological effects of the cAMP-dependent signaling pathway (Conti et al., 1995; Jeremy et al., 1997). Growing evidence supports a role for PKG as an important cGMP target that promotes cGMP effects in cardiac myocytes (Murthy, 2001). The activation of PKG can cause decreased levels of intracellular calcium ions (Ca^2+^) and finally result in the relaxation of smooth muscle. With respect to viral infections, this signaling pathway could have a potential role in viral replication (Li et al., 2018). However, the role of this signaling pathway in mast cells following various IAV infection remains poorly understood. Here, the microassays and RT-qPCR successfully confirmed the preferentially expression of PKG in H1N1-infected P815 cells. Our results provide novel information that the cGMP/PKG signaling pathway has an essential role in P815 cells following H1N1 rather than H5N1 or H7N2 infection. We speculate that this signaling pathway may directly influence the replication ability of the H1N1 virus in mast cells or affects the host responses to infection.

### Crucial Role of the HIF-1 Signaling Pathway in P815 Cells Infected by H7N2 Infection

Hypoxia-inducible factor (HIF) transcription factors are commanders of the cellular response to hypoxia and regulate metabolic processes. HIF-1, which includes HIF-1α and HIF-1β, is widely expressed in diverse immune populations including macrophages, neutrophils, dendritic cells, and lymphocytes (Welsh and Powis, 2003; Palazon et al., 2014). Under hypoxic conditions, HIF-1 is activated. HIF-1α is then transferred into the nucleus and heterodimerizes with HIF-1β through phosphorylation by MAPK, followed by active transcription of downstream genes. It can promote the transcription and expression of various target genes such as endothelin-1 (ET-1), vascular endothelial growth factors (VEGF), heme oxygenase-1 (HO-1), erythropoietin (EPO), and Adrenomedullin (ADM). As the primary regulator of oxygen homeostasis, previous studies have demonstrated that HIF-1 is also expressed in mast cells and affects degranulation of these immune cells and expression of inflammatory factors (Liang et al., 2016). Researchers have shown that HIF-1a could contribute to response about *Staphylococcus aureus* infection in mast cell under hypoxia (Mollerherm et al., 2017). However, whether the HIF-1 signaling pathway is involved in mast cells following IAV infection has yet to be explored. Here, our data showed that the HIF-1α signaling pathway with *p* ≤ 0.05 was preferentially and significantly modulated by DEGs induced by H7N2 virus in P815 cells. Interestingly, MAPK was also found in H7N2-infected cells, which confirms the dominant role of the HIF-1 signaling pathway in mast cells during H7N2 infection. RT-qPCR results also confirmed that the H7N2 virus leads to the high gene expression levels of HIF-1α among the three subtypes of IAVs, indicating the potential role of HIF-1α in P815 cells during H7N2 infection. Previous studies have also demonstrated that HIFs directly regulate the expression of hundreds of genes in mammalian cells (Perez-Perri et al., 2011). Here, further analysis indicated more genes were upregulated in cells following H7N2 virus infection at 12 h. Therefore, we demonstrated for the first time that H7N2 rather than H1N1 or H5N1 infection can trigger the HIF-1 signaling pathway. We speculate that the activation of this signaling pathway is an essential factor that results in the lowest replication of H7N2 in mast cells among the three subtypes of IAV.

### Exosomes Are Preferentially Secreted From H1N1 or H7N2-Infected P815 Cells

Exosomes are vesicles composed of proteins, lipids, and nucleic acids that are surrounded by a bilayer membrane of phospholipids. As an intercellular communicator, exosomes can be secreted by almost all cell types, and are widely involved in the transmission of intercellular substances and information. They play an important role in physiological and pathological processes such as intercellular communication, immune response, tumorigenesis, and development (Raposo and Stoorvogel, 2013). To date, exosomes have been widely used in the diagnosis and treatment of diseases due to their unique biological structure and function such as their small size (50–150 nm) and good biocompatibility (Atay et al., 2018; Liao et al., 2019). With respect to viral infections, exosomes have been reported to influence the infection of viruses in several cells (Longatti et al., 2015). Notably, the components and functions of exosomes differ between the cell types. Studies have demonstrated that exosomes secreted from dendritic cells contain major histocompatibility complex (MHC) molecules and positively activate the immune response of T cells and thus play an important role in immune regulation (Denzer et al., 2000). However, the presence and role of exosomes in mast cells following IAV infection are poorly understood. In our study, much to our surprise, we determined that exosomes were preferentially present in P815 cells infected by H1N1 or H7N2 rather than H5N1. It appeared that the H5N1 virus could not significantly prompt the secretion of exosomes from P815 cells. We speculate that these exosomes from mast cells are pivotal in triggering the robust innate immunity of cells to fight H1N1 or H7N2 infection and effectively eliminate the viruses. To our knowledge, we are the first to report the initial discovery of exosomes in mast cells following H1N1 or H7N2 infection. It remains to be explored what components of exosomes play an essential role in mast cells during IAV infection and how they function.

## Conclusions

We investigated the more profound genomic profiles for the first time and describe the dynamic change of genomes regulated by different subtypes of IAV in mouse mast cells using microassays. Compared with any two of the three IAV-infected groups, DEGs, cellular functions, and signaling pathways were confirmed in the H1N1 and H7N2 groups, and the H7N2 group showed the highest levels. However, few DEGs were detected and various cellular functions and signaling pathways were dramatically suppressed in the H5N1 group. Comparison of the functional annotations and bioinformatic characterization of DEGs in P815 cells infected with three different viruses at the same time could show that histone family genes play a pivotal role in regulated signaling pathways. With an in-depth study on the H1N1 and H7N2 groups, we demonstrated the essential roles of the 5-HT signaling pathway and cGMP/PKG signaling pathway, which were preferentially activated in P815 cells infected by H1N1. We also demonstrated the crucial role of the HIF-1 signaling pathway, which was preferentially activated in P815 cells infected by the H7N2 virus. Furthermore, RT-qPCR results showed significantly increased mRNA levels of 5-HT and PKG in H1N1-infected P815 cells and increased HIF-1 levels in H7N2-infected P815 cells. Exosomes were also preferentially secreted from H1N1-infected or H7N2-infected P815 cells and are potentially pivotal in innate immunity to fight IAV infection. This study provides novel information and insight into the distinct molecular mechanism of H1N1, H5N1, and H7N2 viruses in mast cells from the perspective of genomic profiles.

## Data Availability

The microarray data can be found in the NCBI GEO repository using accession number GSE129623.

## Author Contributions

CH, DM and YXH carried out experiments, analyzed data and wrote the paper. JX, SZ, MW, PQ, HT, and YH designed the study and supervised the project. HW assisted in the data analysis and discussion. CH, HW and YH drew the figures. All authors read and approved the final manuscript.

### Conflict of Interest Statement

The authors declare that the research was conducted in the absence of any commercial or financial relationships that could be construed as a potential conflict of interest.

## Footnotes

^1^https://david.ncifcrf.gov/

^2^http://string-db.org/

## References

[ref1] Abraham S. N. St John A. L. (2010). Mast cell-orchestrated immunity to pathogens. Nat. Rev. Immunol. 10, 440–452. 10.1038/nri2782, PMID: 20498670 PMC4469150

[ref2] Atay S. Wilkey D. W. Milhem M. Merchant M. Godwin A. K. (2018). Insights into the proteome of gastrointestinal stromal tumors-derived exosomes reveals new potential diagnostic biomarkers. Mol. Cell. Proteomics 17, 495–515. 10.1074/mcp.RA117.000267, PMID: 29242380 PMC5836374

[ref3] Benditt E. P. Wong R. L. Arase M. Roeper E. (1955). 5-Hydroxytryptamine in mast cells. Proc. Soc. Exp. Biol. Med. 90, 303–304. 10.3181/00379727-90-22016, PMID: 13273431

[ref4] Boyce J. A. (2007). Mast cells and eicosanoid mediators: a system of reciprocal paracrine and autocrine regulation. Immunol. Rev. 217, 168–185. 10.1111/j.1600-065X.2007.00512.x, PMID: 17498059

[ref5] Cameron C. M. Cameron M. J. Bermejo-Martin J. F. Ran L. Xu L. Turner P. V.. (2008). Gene expression analysis of host innate immune responses during lethal H5N1 infection in ferrets. J. Virol. 82, 11308–11317. 10.1128/JVI.00691-08, PMID: 18684821 PMC2573250

[ref6] Conti M. Nemoz G. Sette C. Vicini E. (1995). Recent progress in understanding the hormonal regulation of phosphodiesterases. Endocr. Rev. 16, 370–389. 10.1210/edrv-16-3-370, PMID: 7671852

[ref7] da Silva E. Z. Jamur M. C. Oliver C. (2014). Mast cell function: a new vision of an old cell. J. Histochem. Cytochem. 62, 698–738. 10.1369/0022155414545334, PMID: 25062998 PMC4230976

[ref8] Denzer K. van Eijk M. Kleijmeer M. J. Jakobson E. de Groot C. Geuze H. J. (2000). Follicular dendritic cells carry MHC class II-expressing microvesicles at their surface. J. Immunol. 165, 1259–1265. 10.4049/jimmunol.165.3.1259, PMID: 10903724

[ref9] Dimitrov N. Atanasova D. Tomov N. Sivrev D. Lazarov N. (2017). Acupuncture causes serotonin release by mast cells. Romanian J. Morphol. Embryol. 58, 961–968. PMID: 29250675

[ref10] Fouchier R. A. Munster V. Wallensten A. Bestebroer T. M. Herfst S. Smith D.. (2005). Characterization of a novel influenza A virus hemagglutinin subtype (H16) obtained from black-headed gulls. J. Virol. 79, 2814–2822. 10.1128/JVI.79.5.2814-2822.2005, PMID: 15709000 PMC548452

[ref11] Galli S. J. Borregaard N. Wynn T. A. (2011). Phenotypic and functional plasticity of cells of innate immunity: macrophages, mast cells and neutrophils. Nat. Immunol. 12, 1035–1044. 10.1038/ni.2109, PMID: 22012443 PMC3412172

[ref12] Galli S. J. Nakae S. Tsai M. (2005). Mast cells in the development of adaptive immune responses. Nat. Immunol. 6, 135–142. 10.1038/ni1158, PMID: 15662442

[ref13] Gusev Y. (2008). Computational methods for analysis of cellular functions and pathways collectively targeted by differentially expressed microRNA. Methods 44, 61–72. 10.1016/j.ymeth.2007.10.005, PMID: 18158134

[ref14] Henz B. M. Maurer M. Lippert U. Worm M. Babina M. (2001). Mast cells as initiators of immunity and host defense. Exp. Dermatol. 10, 1–10. 10.1034/j.1600-0625.2001.100101.x, PMID: 11168574

[ref15] Hu Y. Jin Y. Han D. Zhang G. Cao S. Xie J.. (2012). Mast cell-induced lung injury in mice infected with H5N1 influenza virus. J. Virol. 86, 3347–3356. 10.1128/JVI.06053-11, PMID: 22238293 PMC3302317

[ref16] Huo C. Xiao K. Zhang S. Tang Y. Wang M. Qi P.. (2018). H5N1 influenza a virus replicates productively in pancreatic cells and induces apoptosis and pro-inflammatory cytokine response. Front. Cell. Infect. Microbiol. 8:386. 10.3389/fcimb.2018.00386, PMID: 30460207 PMC6232254

[ref17] Jeremy J. Y. Ballard S. A. Naylor A. M. Miller M. A. Angelini G. D. (1997). Effects of sildenafil, a type-5 cGMP phosphodiesterase inhibitor, and papaverine on cyclic GMP and cyclic AMP levels in the rabbit corpus cavernosum *in vitro*. Br. J. Urol. 79, 958–963. 9202566 10.1046/j.1464-410x.1997.00206.x

[ref18] Kanehisa M. Araki M. Goto S. Hattori M. Hirakawa M. Itoh M.. (2008). KEGG for linking genomes to life and the environment. Nucleic Acids Res. 36, D480–D484. 10.1093/nar/gkm882, PMID: 18077471 PMC2238879

[ref19] Li C. Li L. Jin L. Yuan J. (2018). Heme oxygenase-1 inhibits spring viremia of carp virus replication through carbon monoxide mediated cyclic GMP/protein kinase G signaling pathway. Fish Shellfish Immunol. 79, 65–72. 10.1016/j.fsi.2018.05.014, PMID: 29753142

[ref20] Liang X. Yin G. Ma Y. Xu K. Liu J. Li J. (2016). The critical role of mast cell-derived hypoxia-inducible factor-1alpha in regulating mast cell function. J. Pharm. Pharmacol. 68, 1409–1416. 10.1111/jphp.12622, PMID: 27671226

[ref21] Liao W. Du Y. Zhang C. Pan F. Yao Y. Zhang T.. (2019). Exosomes: the next generation of endogenous nanomaterials for advanced drug delivery and therapy. Acta Biomater. 86, 1–14. 10.1016/j.actbio.2018.12.045, PMID: 30597259

[ref22] Liu B. Meng D. Wei T. Zhang S. Hu Y. Wang M. (2014). Apoptosis and pro-inflammatory cytokine response of mast cells induced by influenza A viruses. PLoS One 9:e100109. 10.1371/journal.pone.0100109, PMID: 24923273 PMC4055757

[ref23] Lohmann S. M. Vaandrager A. B. Smolenski A. Walter U. De Jonge H. R. (1997). Distinct and specific functions of cGMP-dependent protein kinases. Trends Biochem. Sci. 22, 307–312. 10.1016/S0968-0004(97)01086-4, PMID: 9270304

[ref24] Longatti A. Boyd B. Chisari F. V. (2015). Virion-independent transfer of replication-competent hepatitis C virus RNA between permissive cells. J. Virol. 89, 2956–2961. 10.1128/JVI.02721-14, PMID: 25505060 PMC4325714

[ref25] Marshall J. S. (2004). Mast-cell responses to pathogens. Nat. Rev. Immunol. 4, 787–799. 10.1038/nri1460, PMID: 15459670

[ref26] Marshall J. S. King C. A. McCurdy J. D. (2003). Mast cell cytokine and chemokine responses to bacterial and viral infection. Curr. Pharm. Des. 9, 11–24. 10.2174/1381612033392413, PMID: 12570671

[ref27] Meng D. Huo C. Wang M. Xiao J. Liu B. Wei T.. (2016). Influenza a viruses replicate productively in mouse Mastocytoma cells (P815) and trigger pro-inflammatory cytokine and chemokine production through TLR3 Signaling pathway. Front. Microbiol. 7:2130. 10.3389/fmicb.2016.02130, PMID: 28127293 PMC5226950

[ref28] Mollerherm H. Branitzki-Heinemann K. Brogden G. Elamin A. A. Oehlmann W. Fuhrmann H.. (2017). Hypoxia modulates the response of mast cells to *Staphylococcus aureus* infection. Front. Immunol. 8:541. 10.3389/fimmu.2017.00541, PMID: 28553287 PMC5425595

[ref29] Mossner R. Lesch K. P. (1998). Role of serotonin in the immune system and in neuroimmune interactions. Brain Behav. Immun. 12, 249–271. 10.1006/brbi.1998.0532, PMID: 10080856

[ref30] Murthy K. S. (2001). Activation of phosphodiesterase 5 and inhibition of guanylate cyclase by cGMP-dependent protein kinase in smooth muscle. Biochem. J. 360, 199–208. 10.1042/bj3600199, PMID: 11696008 PMC1222218

[ref31] Palazon A. Goldrath A. W. Nizet V. Johnson R. S. (2014). HIF transcription factors, inflammation, and immunity. Immunity 41, 518–528. 10.1016/j.immuni.2014.09.008, PMID: 25367569 PMC4346319

[ref32] Perez-Perri J. I. Acevedo J. M. Wappner P. (2011). Epigenetics: new questions on the response to hypoxia. Int. J. Mol. Sci. 12, 4705–4721. 10.3390/ijms12074705, PMID: 21845106 PMC3155379

[ref33] Raposo G. Stoorvogel W. (2013). Extracellular vesicles: exosomes, microvesicles, and friends. J. Cell Biol. 200, 373–383. 10.1083/jcb.201211138, PMID: 23420871 PMC3575529

[ref34] Sugiura N. Uda A. Inoue S. Kojima D. Hamamoto N. Kaku Y.. (2011). Gene expression analysis of host innate immune responses in the central nervous system following lethal CVS-11 infection in mice. Jpn. J. Infect. Dis. 64, 463–472. PMID: 22116324

[ref35] Tisoncik J. R. Korth M. J. Simmons C. P. Farrar J. Martin T. R. Katze M. G. (2012). Into the eye of the cytokine storm. Microbiol. Mol. Biol. Rev. 76, 16–32. 10.1128/MMBR.05015-11, PMID: 22390970 PMC3294426

[ref36] Tong S. Zhu X. Li Y. Shi M. Zhang J. Bourgeois M.. (2013). New world bats harbor diverse influenza A viruses. PLoS Pathog. 9:e1003657. 10.1371/journal.ppat.1003657, PMID: 24130481 PMC3794996

[ref37] Vasin A. V. Temkina O. A. Egorov V. V. Klotchenko S. A. Plotnikova M. A. Kiselev O. I. (2014). Molecular mechanisms enhancing the proteome of influenza A viruses: an overview of recently discovered proteins. Virus Res. 185, 53–63. 10.1016/j.virusres.2014.03.015, PMID: 24675275

[ref38] Welsh S. J. Powis G. (2003). Hypoxia inducible factor as a cancer drug target. Curr. Cancer Drug Targets 3, 391–405. 10.2174/1568009033481732, PMID: 14683498

[ref39] Welton J. L. Khanna S. Giles P. J. Brennan P. Brewis I. A. Staffurth J.. (2010). Proteomics analysis of bladder cancer exosomes. Mol. Cell. Proteomics 9, 1324–1338. 10.1074/mcp.M000063-MCP201, PMID: 20224111 PMC2877990

[ref40] Wu H. Denna T. H. Storkersen J. N. Gerriets V. A. (2019). Beyond a neurotransmitter: the role of serotonin in inflammation and immunity. Pharmacol. Res. 140, 100–114. 10.1016/j.phrs.2018.06.015, PMID: 29953943

[ref41] Wu M. L. Xu D. S. Bai W. Z. Cui J. J. Shu H. M. He W.. (2015). Local cutaneous nerve terminal and mast cell responses to manual acupuncture in acupoint LI4 area of the rats. J. Chem. Neuroanat. 68, 14–21. 10.1016/j.jchemneu.2015.06.002, PMID: 26148746

